# Dietetic Economies

**Published:** 1856-11

**Authors:** 


### DIETETIC ECONOMIES.

As winter is approaching with its pinchings of the poor, it may be well for many to study, what articles of food are the most nutritious and cheapest; that is, what kinds of food will go farthest for the least money. Not a few in our large cities lay the foundation of incurable and fatal diseases, by being stinted in their food, and who would not have been stinted, had they expended what money they had in the most judicious manner. The ignorance and inconsiderateness of the poor is sometimes amazing. The ladies of the Widows' Aid Society, who do so much for humanity every winter, have found it expedient to refuse giving money to any of their beneficiaries, but ascertain their actual wants, and give them orders for such articles of food as are deemed best. They found that when money was given, it would be expended for tea and coffee, and fine flour for the luxuries instead of the necessaries of life. We trust the following table may be of practical advantage to this humane society, as well as to many poor, and prudent and worthy families in this, and other large cities and towns. We believe a man feels as happy after a plain dinner, as after a luxurious one; certain are we, that he sleeps the sounder that night and feels the better for it all next day; all the advantage to the luxurious liver, is in the transient passage down the throat.

| | |
|---|---|
| 1 lb. Cucumbers, at — per doz., yields — per cent of nutriment, | 2 1-2 |
| " Melons, | 3 |
| " Turnips, | 4 1-2 |
| " Cabbage, | 7 1-2 |
| " Carrots, | 10 |
| " Beets, | 15 |
| " Apples, | 16 |
| " Peaches, | 20 |
| " Potatoes, at 75c. per bus. or 1 1-4c. per lb. | 22 1-2 |
| " Cherries, | 25 |
| " Grapes, | 27 |
| " Plums, | 29 |
| " Oat Meal, at $4 per cwt. or 4c. per lb. | 75 |
| " Rye Flour, at 7 per bbl. or 4c. per lb. | 79 |
| " Rice, . - 5 per cwt. or 5c. per lb. | 86 |
| " Barley Meal 3 per cwt. or 3c. per lb. | 88 |
| " Wheat Flour, 10 per bbl. or 5c. per lb. | 90 |
| " Corn Meal, 3 per cwt. or 3 1-2 " " | 91 |
| " White Beans, 2 per bus. or 4 1-2 " " | 95 |

As to the blanks above, any housekeeper can weigh the articles, and by comparing the price per bushel or dozen, with the amount of nutriment yielded, can determine at once, the relative value as a food. But it will be seen at once, that white beans, whole or split peas, hominy, oat meal, corn meal, samp, hulled corn, crushed wheat, rice, are among the cheapest, most wholesome and most nutritious articles of food, and are alike recommended to those who want to be economical, and those who want to be healthy. If fruits were largely used with the above diet, either baked, if green, or stewed when dried, both the digestion and health would be greatly improved, to say nothing of the agreeableness of the addition. Not one person in a thousand has any adequate idea of the value of fruits as an article of diet. A thousand bushels of grapes and apples should be grown where one now is, especially as considering the outlay and labor, they are the most profitable of all crops.

